# CAR-Ts redirected against the Thomsen-Friedenreich antigen CD176 mediate specific elimination of malignant cells from leukemia and solid tumors

**DOI:** 10.3389/fimmu.2023.1219165

**Published:** 2023-10-17

**Authors:** Anna Christina Dragon, Luca Marie Beermann, Melina Umland, Agnes Bonifacius, Chiara Malinconico, Louisa Ruhl, Patrik Kehler, Johanna Gellert, Lisa Weiß, Sarah Mayer-Hain, Katharina Zimmermann, Sebastian Riese, Felicitas Thol, Gernot Beutel, Britta Maecker-Kolhoff, Fumiichiro Yamamoto, Rainer Blasczyk, Axel Schambach, Michael Hust, Michael Hudecek, Britta Eiz-Vesper

**Affiliations:** ^1^ Institute of Transfusion Medicine and Transplant Engineering, Hannover Medical School (MHH), Hannover, Germany; ^2^ Glycotope GmbH, Berlin, Germany; ^3^ Institute of Experimental Hematology, Hannover Medical School (MHH), Hannover, Germany; ^4^ Department of Hematology, Hemostasis, Oncology and Stem Cell Transplantation, Hannover Medical School (MHH), Hannover, Germany; ^5^ Department of Pediatric Hematology and Oncology, Hannover Medical School (MHH), Hannover, Germany; ^6^ Josep Carreras Leukaemia Research Institute (IJC), Badalona, Spain; ^7^ Department of Medical Biotechnology, Technical University of Braunschweig, Braunschweig, Germany; ^8^ Department of Internal Medicine II, University Hospital of Würzburg, Wuerzburg, Germany

**Keywords:** CD176, Thomsen-Friedenreich antigen, pan-tumor antigen, carbohydrate antigen, CAR-T cell therapy, immunotherapy, cancer, solid tumors

## Abstract

**Introduction:**

Chimeric antigen receptor-engineered T cells (CAR-Ts) are investigated in various clinical trials for the treatment of cancer entities beyond hematologic malignancies. A major hurdle is the identification of a target antigen with high expression on the tumor but no expression on healthy cells, since “on-target/off-tumor” cytotoxicity is usually intolerable. Approximately 90% of carcinomas and leukemias are positive for the Thomsen-Friedenreich carbohydrate antigen CD176, which is associated with tumor progression, metastasis and therapy resistance. In contrast, CD176 is not accessible for ligand binding on healthy cells due to prolongation by carbohydrate chains or sialylation. Thus, no “on-target/off-tumor” cytotoxicity and low probability of antigen escape is expected for corresponding CD176-CAR-Ts.

**Methods:**

Using the anti-CD176 monoclonal antibody (mAb) Nemod-TF2, the presence of CD176 was evaluated on multiple healthy or cancerous tissues and cells. To target CD176, we generated two different 2^nd^ generation CD176-CAR constructs differing in spacer length. Their specificity for CD176 was tested in reporter cells as well as primary CD8^+^ T cells upon co-cultivation with CD176^+^ tumor cell lines as models for CD176^+^ blood and solid cancer entities, as well as after unmasking CD176 on healthy cells by vibrio cholerae neuraminidase (VCN) treatment. Following that, both CD176-CARs were thoroughly examined for their ability to initiate target-specific T-cell signaling and activation, cytokine release, as well as cytotoxicity.

**Results:**

Specific expression of CD176 was detected on primary tumor tissues as well as on cell lines from corresponding blood and solid cancer entities. CD176-CARs mediated T-cell signaling (NF-κB activation) and T-cell activation (CD69, CD137 expression) upon recognition of CD176^+^ cancer cell lines and unmasked CD176, whereby a short spacer enabled superior target recognition. Importantly, they also released effector molecules (e.g. interferon-γ, granzyme B and perforin), mediated cytotoxicity against CD176^+^ cancer cells, and maintained functionality upon repetitive antigen stimulation. Here, CD176L-CAR-Ts exhibited slightly higher proliferation and mediator-release capacities. Since both CD176-CAR-Ts did not react towards CD176^-^ control cells, their response proved to be target-specific.

**Discussion:**

Genetically engineered CD176-CAR-Ts specifically recognize CD176 which is widely expressed on cancer cells. Since CD176 is masked on most healthy cells, this antigen and the corresponding CAR-Ts represent a promising approach for the treatment of various blood and solid cancers while avoiding “on-target/off-tumor” cytotoxicity.

## Graphical Abstract

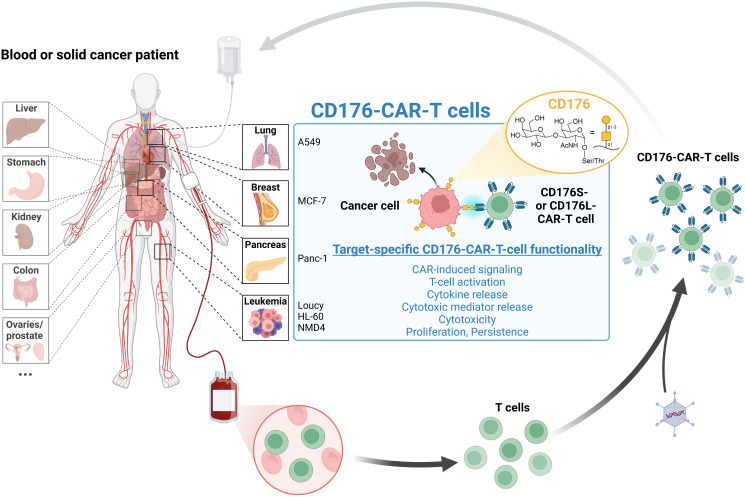

## 1 Introduction

CAR-Ts widely emerged as valuable tool to selectively target and eliminate malignant cells in context of different diseases. Especially CAR-Ts targeting CD19 or B-cell maturation antigen (BCMA) mediated remarkable overall response rates of up to 92% (1) in patients where former treatments were not curative or who suffered from relapses (2), and were shown to persist decade-long in the patients (3). To date, four CD19- and two BCMA-CAR-T products are approved for the treatment of different late-stage hematological malignancies by FDA and EMA. As drawback, successful therapy with CD19- or BCMA-targeting CAR-Ts is often characterized by persistent depletion of healthy B-lineage cells resulting in hypogammaglobulinemia (4). Whereas B-cell aplasia can be compensated by intermittent infusion of pooled immunoglobulins (5), elimination of healthy cells in other cancer entities usually cannot be compensated (6, 7). Also referred to as “on-target/off-tumor” cytotoxicity, immune-mediated attacks on healthy cells expressing the same antigen therefore underscore the importance of finding targets exclusively expressed on malignant cells. Furthermore, in CD19-targeted CAR-T therapies, it has also been shown that 30-70% of relapses after CAR-T administration are due to downregulation or loss of CD19 antigen (8). Therefore, another major hurdle is the choice of an antigen with low probability of antigen escape.

The disaccharide CD176 is a carbohydrate tumor antigen with prominent cancer specificity. Also known as α-anomeric configuration of Thomsen-Friedenreich (TFα) antigen or core-1, it is defined as the carbohydrate epitope sequence Galβ1-3GalNAcα1-R linked to serine or threonine residues of different carrier glycoproteins (9). With an estimated expression on about 90% of various carcinomas (10), such as breast (86%), colon (60-70%), gastric (69%), prostate (56%), lung (48%), liver (38%) and kidney (15%) cancers (reviewed in 9), as well as different leukemias and lymphomas (11), it is regarded as a pan-tumor antigen. In healthy tissues, CD176 is as precursor core-1 part of the O-linked glycosylation cascade. Thus, after transport of carrier proteins to the cell membrane, terminal CD176 is virtually absent on the surface of healthy human adult cells due to its masking by prolonged carbohydrate chains or sialylation. An extensive study by Cao et al. confirmed absence of CD176 in a wide panel of cells from various healthy adult tissues (12).

The mechanisms by which CD176 is exposed on tumor cells are complex and not completely clear, but detection of CD176 has been shown to correlate with tumor progression, invasive and metastatic cancer properties, as well as a less favorable prognosis in different tumor entities (9). For example, a study evaluating 264 patients with primary colorectal cancer showed a significantly decreased survival probability of patients with CD176-positive tumors (13). In line, CD176 was found to be widely expressed on lung, breast and liver cancer-initiating cells (14), as well as quiescent chronic myeloid leukemia (CML) stem cells (15), all of which are not approachable with conventional therapies and often responsible for therapeutic resistance and disease progression.

Thus, the prominent and cancer-specific expression of CD176 as well as its critical role in several malignant processes led to studies on its potential use as a target for vaccination or treatment with mAbs. Yi et al. successfully showed that treatment of CD176^+^ leukemic cells KG1 and MT4 with a CD176 antiserum induced apoptosis (16, 17). In addition, Tati et al. described the humanized mAb hJAA-F11, which induced significant cytotoxicity towards CD176^+^ breast and lung cancer cell lines such as A549 (18). *In vivo*, treatment with hJAA-F11 reduced tumor growth in a mouse xenograft triple-negative CD176^+^ breast cancer model (19).

Based on the above facts, we postulate that CD176 presents an ideal target for CAR-T approaches against various cancers with expected low “on-target/off-tumor” cytotoxicity and low likelihood of antigen escape due to its tumor-promoting properties. Here, we report the development of two 2^nd^-generation CD176-CARs based on the CD176-specific mAb Nemod-TF2 (9). The potency and functionality of CD176-CAR-Ts with a short (CD176S-CAR) and a long (CD176L-CAR) extracellular spacer domain was investigated to determine the configuration for optimal binding distance to target cell epitopes on cancer cells with potentially different CD176 carrier proteins. CD176-CAR-Ts were specifically activated, cytotoxic, proliferated and released cytokines and cytotoxic mediators after co-cultivation with CD176^+^ target cells from different cancer entities, while they did not react against CD176^-^ target cells. Whereas the CD176S-CAR initiated signaling towards a wider selection of CD176^+^ cells from different tumor entities, CD176L-CAR-Ts showed slightly higher proliferation and mediator release capacities upon repetitive antigen stimulation.

Our data provide conclusive evidence and show for the first time that the TF antigen CD176 can be targeted by CAR-Ts. CD176-CAR-T therapy can provide specific treatment for multiple blood and solid cancers while circumventing common CAR-associated challenges such as “on-target/off-tumor” cytotoxicity or antigen escape.

## 2 Materials and methods

### 2.1 Human sample materials

All experiments were performed using residual blood samples from routine platelet collection at the Institute of Transfusion Medicine and Transplant Engineering, Hannover Medical School. According to standard donation requirements, the respective donors had no signs of acute infection and no previous history of blood transfusion. Written informed consent was obtained from all donors as approved by the Ethics Committee of Hannover Medical School (2519–2014, 3639–2017). Peripheral blood mononuclear cells (PBMCs) were isolated by density gradient centrifugation using Lymphosep (c.c.pro).

### 2.2 Immunohistochemistry

For immunohistochemistry stainings, FDA standard tissue array and tumor tissue array (Tissue Array) were deparaffinized and rehydrated in a descending alcohol series before antigen unmasking with TrisEDTA (pH 9.0). Endogenous peroxidase was blocked using 3% H_2_O_2_ followed by non-specific binding block with BlockAid (Invitrogen). The tissues were then stained with Nemod-TF2 (Glycotope) and Envision Flex (Agilent), and the color developed using DAB+ staining solution (Agilent). All slides were counterstained using Mayer’s Haematoxylin (Thermo Fisher Scientific) and mounted in Entellan (Merck). Slides were photographed using VS200 Research Slide Scanner (Olympus Lifescience) and evaluated by two independent analysts to determine the immunoreactive score (IRS, score range 0-12) (20) for presence of membranous CD176 on tumor and H-Score (score range score range 0-300) (21) for presence on normal tissues.

### 2.3 Construction of CD176-CAR vectors

The single-chain variable fragment (scFv) for the CD176-CAR was synthesized (Thermo Fisher Scientific) based on the humanized sequences of the anti-CD176 mAb Nemod-TF2 (9) (Glycotope). scFv heavy and light chains were connected by a (G_4_S)_3_ linker and cloned into the previously described TÜ165-CAR-epHIV7 vectors by using NheI and RsrII restriction sites to replace the TÜ165 scFv (22). Two different CAR constructs with either a “hinge-only” (12 amino acids, aa) short-spacer domain (CD176S-CAR) or a “hinge-CH2-CH3” (229aa) long-spacer domain derived from IgG4-Fc (CD176L-CAR) were generated. The spacer domain was followed by a CD28-derived transmembrane domain as well as 4-1BB and CD3ζ cytoplasmic signaling domains. Following a T2A ribosomal skip element, a truncated epidermal growth factor receptor (EGFRt) sequence (23) serving as marker for detection and enrichment of transduced cells, was integrated. As control CAR, a CD19 CAR with a “hinge-only” short-spacer domain was generated analogously by replacing the TÜ165 scFv from the previously described TÜ165-CAR-epHIV7 vector (22) with an FMC63-derived scFv. In that case, scFv heavy and light chains were connected by using a Whitlow linker (24).

### 2.4 Generation and titration of lentiviral particles

CD176S- and CD176L-CAR lentiviral particles were produced using the calcium phosphate method as described before (22) and explained in **Supplementary Methods**.

### 2.5 Reporter assay to determine CD176-CAR signaling

A previously described reporter cell line based on the Jurkat T-cell line JE6-1 (25) was transduced with lentiviral particles (multiplicities of infections, MOI = 3) under addition of 5μg/mL Polybrene (Merck) using spinoculation. Recognition of unmasked CD176 was assessed by prior treatment with vibrio cholerae neuraminidase (VCN; Merck) for 48h. Recognition of CD176^-^ and CD176^+^ target cells was assessed by co-culturing with respective target cell lines previously labelled with CellTrace™ violet proliferation dye (CTV; Thermo Fisher Scientific) for 24h in an effector to target (E:T) ratio of 1:1. CD176-CAR signaling was determined by measurement of enhanced cyan fluorescent protein (eCFP) reporting for nuclear factor kappa-light-chain-enhancer of activated B cells (NF-κB) activation in EGFRt^+^ JE6-1 cells by flow cytometry. As positive control, transduced cells were activated by anti-CD3/CD28 beads (Thermo Fisher Scientific).

### 2.6 Generation of primary CD176-CAR-Ts

CD8^+^ T cells were isolated (Miltenyi Biotec) from PBMCs of healthy donors and activated with anti-CD3/CD28 beads at a ratio of 1:1. In accordance to a widely-used CliniMACS Prodigy^®^ protocol for GMP-compliant manufacturing of CAR-Ts for the clinical use (26), they were cultured in TexMACS (Miltenyi Biotec) with 3% human serum (c.c.pro; CTL medium) supplemented with 12.5ng/ml interleukin (IL)-7 and IL-15 (PeproTech). After one day, T cells were transduced with lentiviral particles (MOI 3) by spinoculation in presence of 5μg/mL Polybrene. The beads were removed on day 2 and cells were split according to their growth approximately every second day. On day 8-9, EGFRt^+^ T cells were enriched by using biotinylated anti-EGFR antibody and anti-biotin microbeads (Miltenyi Biotec). EGFRt^+^ cells were expanded until day 12-14. Untransduced T cells were treated equally during the whole process and served as control in all experiments. For some experiments, CD19-CAR-Ts were generated using the same transduction, enrichment and expansion protocol and served as additional controls.

### 2.7 Evaluation of CD176-CAR-T functionality

To determine functionality of CD176-CAR-Ts, they were co-cultured with CD176^-^ and CD176^+^ target cells in CTL medium for the indicated time periods. For evaluation of proliferation, cytokine release and phenotype following repetitive antigen stimulation, frozen CD176-CAR-Ts were thawed one day before the co-culture and re-stimulated with the same number of target cells after 3/4, 7/8 and 10/11 days.

### 2.8 Flow cytometry

Antibodies used for flow cytometry are listed in **Table S3**. Samples were analyzed on a 10-color BD FACSCanto Flow Cytometer (Becton Dickinson). Data were analyzed using FlowJo V10.

### 2.9 Multiplex cytokine analysis

To determine effector molecule concentrations in co-culture supernatants, the LEGENDplex Human CD8/NK Panel (BioLegend) was performed according to the manufacturer’s instructions. Data were analyzed with LEGENDplex V.8.0 software (BioLegend). Target-induced release of granzyme B, perforin and granulysin was calculated by subtraction of concentrations in the co-cultures by the respective (CAR-)T cells cultured alone.

### 2.10 Cytotoxicity

Killing capacity of CAR-Ts was assessed by co-cultivation with CTV-labelled target cells (E:T ratio 2:1) for 48h. Afterwards, 7-AAD (BioLegend) was used to discriminate dead CTV^+^ cells via flow cytometry. For the evaluation of cytotoxicity in co-cultures of CD176-CAR-Ts with Panc-1 cells for 4 days, transmitted-light microscope images were taken with an Olympus IX81 microscope combined with a digital B/W camera using 10x objective lenses and analyzed with Xcellence Pro image software (all from Olympus). Representative pictures are shown.

### 2.11 Statistical analysis

Statistical analysis was performed by Graph Pad Prism V9.1.1 using two-way ANOVA and Tukey’s or Šídák’s multiple comparisons tests. ns: not significant (p>0.05), *p ≤ 0.05, **p ≤ 0.01, ***p ≤ 0.001.

## 3 Results

### 3.1 Specific expression of CD176 on malignant cells from different blood and solid cancers

**Figure 1:**
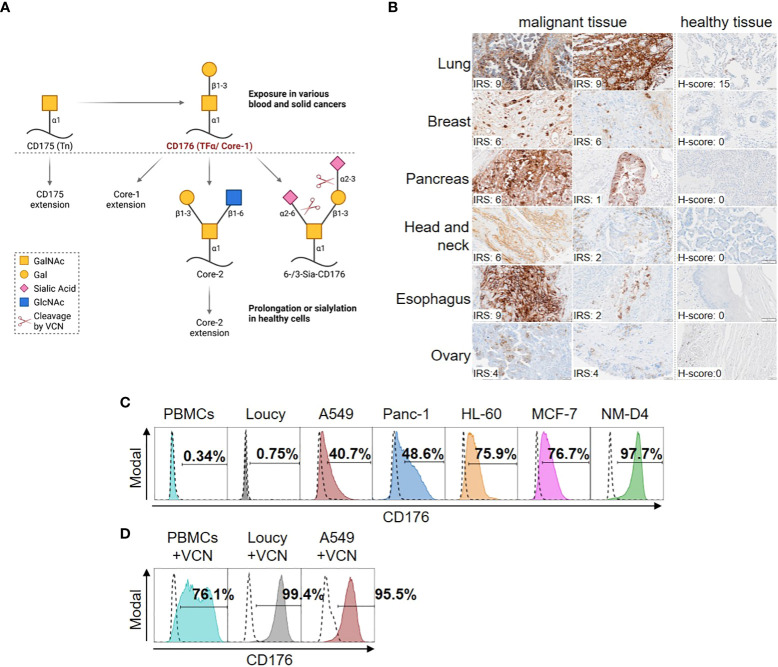
Selective expression of CD176 on malignant cells from different blood and solid cancers. **(A)** CD176 is a disaccharide composed of galactose (Gal) and N-acetylgalactosamine (GalNAc) linked to serine or threonine of different carrier proteins in an α-anomeric configuration. Also known as core-1, CD176 is generated from CD175 (also known as T-nouvelle or Tn) as part of the O-linked glycosylation cascade in the golgi-apparatus. In that, CD176 is elongated to core-2 and further to O-glycans of up to 20 monosaccharides by sequential attachment of carbohydrates, which can, e.g., be terminated with blood group A and B antigens. Otherwise, mono- (6- or 3-Sia-CD176) or di-sialylation (6-/3-Sia-CD176) can terminate the elongation. Thus, only CD176 masked by carbohydrate chain prolongation or terminal sialylation is transported and expressed on the surface of healthy adult cells, whereas it is exposed in various blood and solid cancers. Sia-CD176 can be unmasked to CD176 via hydrolysis of glycosidic linkages of terminal sialic acid using VCN. Created with BioRender.com. **(B)** Tissue samples of healthy donors or patients with different kinds of cancer (healthy tissue array and tumor tissue array) were stained using Nemod-TF2. All slides were counterstained using Mayer’s Haematoxylin and evaluated by two independent analysts to determine the immunoreactive score (IRS) for presence of membranous CD176 on tumor and H-Score to ensure absence on normal tissues. For normal human tissues, CD176 staining was performed on six different normal human tissues (Lung, Breast, Pancreas, Salivary gland, Esophagus and Ovary). Quantification of at least four different donors per organ showed negative signals (H-score below 50) for CD176 in five of the tissues. In two out of seven lung donor samples a positive CD176 staining was detected in the alveolar epithelium. **(C)** Surface expression of CD176 on PBMCs from healthy donors and cell lines from different blood and solid cancer entities detected by staining with Nemod-TF2 in flow cytometry. Positive gates are set on the unstained cells, respectively (dashed lines). **(D)** Detection of CD176 by Nemod-TF2 on PBMCs and cell lines that were treated with VCN for 24h before flow cytometry. **(C, D)** PBMCs and PBMCs+VCN were stained by murine Nemod-TF2 to prevent background staining by anti-human detection reagent on human cells.

Whereas CD176 is elongated as part of the O-linked glycosylation cascade or sialylated (Sia-CD176) on the cell surface of healthy adult cells, it is exposed in most blood and solid cancers (**Figure 1A**). Sia-CD176 can be unmasked to CD176 via hydrolysis of terminal sialic acid using VCN.

To target CD176 expressed on malignant cells, Nemod-TF2, a previously described CD176-specific mAb with high affinity and specificity (9), was evaluated in immunohistochemistry staining (**Figure 1B**) and flow cytometry (**Table 1**, **Figures 1C, D**). In tissue samples from different solid cancer entities, presence of CD176 on the surface of tumor cells was revealed and evaluated by using immunoreactive scores (IRS, scoring system range 0-12 (20)) (**Figure 1B**). Despite heterogeneous expression with IRS scores ranging from 1-9 within samples from different patients, all depicted tumor samples presented CD176, while the corresponding isotype controls showed no signal (**Figure S1A**). Evaluation of corresponding healthy tissues revealed absence of CD176 on the surface with exception of low expression in two out of seven normal lung tissue samples.

**Table 1:** Presence of CD176 on blood samples of T-ALL and B-ALL patients.

	Material	positive patients	positivity range	control
**T-ALL**	first diagnosis	2/6 (33%)	17-34%	healthy PBMCs (n=2)
	relapse	5/9 (56%)	11-68%	healthy PBMCs (n=2)
**B-ALL**	first diagnosis	2/8 (25%)	13-21%	healthy PBMCs (n=3)
	relapse	3/9 (30%)	10-22%	healthy PBMCs (n=3)

Blood samples of T-ALL or B-ALL patients taken at their first diagnosis or upon relapse of the primary tumor were stained for presence ofCD176 by using Nemod-TF2 and flow cytometry. Patients with presence of CD176 on ≥10% of tumor cells were regarded as positive (positivity cut-off). PBMCs isolated from healthy donors (n=2-3) served as negative controls, whereby none of them exceeded the positivity cut-off [data not shown].

Using flow cytometry, CD176 was detected on malignant cells in blood samples from one third of the tested patients with T-cell acute lymphoblastic leukemia (T-ALL) or B-cell acute lymphoblastic leukemia (B-ALL) (**Table 1**). Of note, the frequency of CD176-positive leukemia samples was higher in patients with a relapse than in patient samples of first diagnosis. Expression of CD176 on T cells in a T-ALL patient was moreover restricted to the leukemic precursor T cells (67.9% of CD7^+^CD3^-^ cells), whereas mature CD7^+^CD3^+^ T cells from the same patient were mainly CD176-negative (**Figure S1B**).

As models of identified CD176^+^ cancer entities for the functional assessment of CD176-CAR-Ts, we characterized different epithelial and leukemic cancer cell lines via flow cytometry using Nemod-TF2 (**Figure 1C**). No CD176 was detectable on the T-ALL cell line Loucy (< 1%), which therefore served as a negative control in all further experiments. All other tested cancer cell lines showed intermediate to high frequencies of CD176^+^ cells with 40.7% for A549 (lung adenocarcinoma), 48.6% for Panc-1 (pancreatic epithelioid carcinoma), 75.9% for HL-60 (acute promyelocytic leukemia, APL), 76.7% for MCF-7 (breast adenocarcinoma) and 98.2% for NM-D4 cells (CML). Expression of CD176 was stable during the course of the experiments. Interestingly, following sorting of HL-60 and MCF-7 into a cell fraction with high CD176 expression and a fraction with negative/low CD176 expression, respectively, CD176 expression returned to comparable levels in both populations within few days of cultivation indicating a fluctuating expression (**Figures S1C–E**). As expected, no CD176 was detectable on the surface of PBMCs isolated from healthy donors.

VCN-treatment of CD176^-^ Loucy and intermediate-positive A549 increased detection of terminal CD176 by Nemod-TF2 staining to 99.4% and 95.5% of the cells, respectively (**Figure 1D**). Analogously, VCN treatment of PBMCs from healthy donors enabled CD176 detection on the majority (76.1%) of cells.

### 3.2 CD176-CARs mediate T-cell signaling upon specific recognition of unmasked CD176 or CD176^+^ cell lines from various solid and blood cancer entities

**Figure 2:**
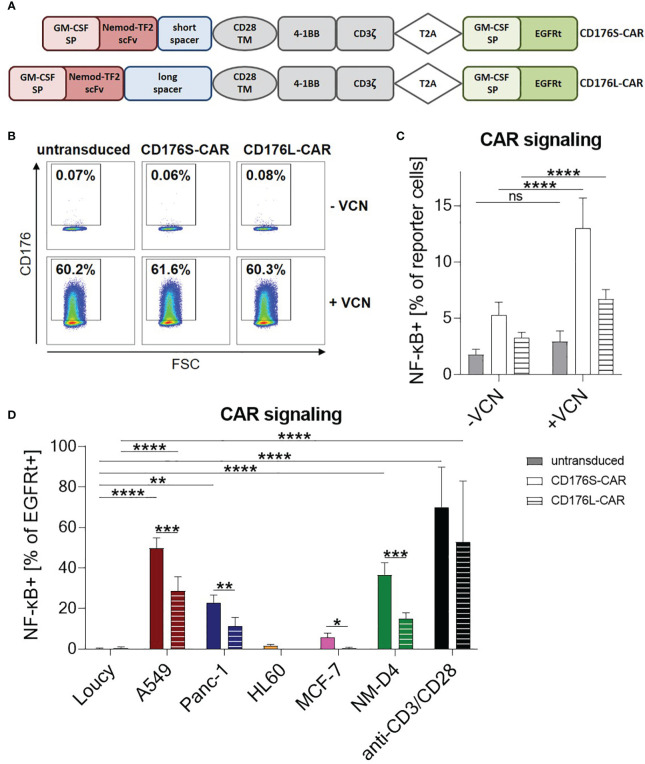
CD176-CARs mediate T-cell signaling upon specific recognition of unmasked CD176 or CD176^+^ cell lines from various solid and blood cancer entities. **(A)** The CD176-CAR constructs consist of a granulocyte macrophage colony-stimulating factor (GM-CSF) signal peptide (SP), a single-chain variable fragment (scFv) based on the humanized version of anti-CD176 antibody Nemod-TF2, an IgG4-Fc spacer of either 12 (CD176S) or 229 amino acids (CD176L), and a CD28 transmembrane domain (TM). 4-1BB and CD3ζ were used as cytoplasmic signaling domains. Via T2A ribosomal skip element, a GM-CSF SP and truncated epidermal growth factor receptor (EGFRt) sequence served as selection marker. **(B, C)** CD176-CAR-transduced reporter cells were treated with VCN (+VCN) or left untreated (-VCN). After two days, **(B)** expression of CD176 shown by representative dot plots and **(C)** NF-κB activation as assessed by an eCFP reporter cassette were measured by flow cytometry (n=11). **(D)** CD176-CAR-transduced reporter cells were co-cultured with the indicated target cells in an E:T ratio of 1:1 for 24h. As positive control, reporter cells were activated with anti-CD3/CD28 antibody-coupled beads. NF-κB activation was evaluated in transduced (EGFRt^+^) cells (n=6; HL-60 n=3). **(C, D)** Data are shown as mean+SD. Statistical analysis was performed using two-way ANOVA and Šídák’s multiple comparisons test **(D)** and is indicated in black for the CD176S-CAR, in grey for the CD176L-CAR and in light grey for comparisons between both constructs. ns: not significant, *p ≤ 0.05, **p ≤ 0.01, ***p ≤ 0.001, ****p ≤ 0.0001.

Two 2^nd^ generation CARs (CD176S and CD176L) differing in their spacer length were generated based on the scFv of the humanized version of Nemod-TF2 (**Figure 2A**). Both constructs comprised signaling domains derived from 4-1BB and CD3ζ.

To test the ability of CD176S- and CD176L-CARs to trigger signal transduction upon recognition of CD176^+^ targets, the constructs were first expressed in a Jurkat T-cell derived reporter cell line (25). These cells, which are naturally CD176-negative, were treated with VCN, resulting in CD176 accessibility on approximately 60% of the cells (**Figures 2B**; **S2**). This unmasking of CD176 on the surface of reporter cells increased NF-κB activation by both, CD176S- and CD176L-CAR constructs. For untransduced reporter cells, NF-κB activity remained nearly identical with and without VCN treatment. Thus, both generated CD176-CARs were able to recognize the CD176 epitope with high specificity. Overall, CD176S- or CD176L-CAR-transduced reporter cells showed minimal background activity of NF-κB compared with untransduced reporter cells (**Figure 2C**).

Reporter cells were further evaluated by co-cultivation with the previously identified CD176^+^ and CD176^-^ cell lines (**Figures 1C**, **2D**). For both constructs, specific upregulation of NF-κB activity following co-cultivation with the CD176^+^ target cell line A549 was observed. Additionally, the CD176S-CAR, and in tendency also the CD176L-CAR, mediated specific NF-κB activation upon recognition of CD176^+^ Panc-1 and NM-D4 cells. Although HL-60 and MCF-7 showed a higher expression of CD176 in comparison to Panc-1 and A549 (**Figure 1C**), they only led to slight activation of the CD176S-CAR and not the CD176L-CAR. Importantly, the CD176^-^ control cell line Loucy did not induce NF-κB activation.

In summary, target recognition was stronger for the CD176S- compared with the CD176L-CAR (average 49.8% vs. 28.75% (A549); 22.7% vs. 11.2% (Panc-1); 36.6% vs. 14.8% (NM-D4)) indicating that the short spacer in the CAR construct enabled superior binding to CD176 present on carrier proteins of all evaluated CD176^+^ cell lines.

### 3.3 CD176-CAR-Ts are activated and release IFN-γ upon recognition of CD176^+^ cell lines from solid and blood cancer entities

CD176S- and CD176L-CAR constructs were transduced into primary CD8^+^ T cells. During expansion of CD176S- and CD176L-CAR-Ts, there was no difference between the cell numbers, activation state, cytokine release and memory phenotype of CD176S- and CD176L-CAR-Ts compared to untransduced T cells cultured with the same protocol (**Figures S3A–C, E, F**). After 9 days of expansion, the T-cell products mainly exhibited a central memory CD8^+^ T cell phenotype (TCM; CD45RO^+^/CCR7^+^, 78-81%). The frequency of transduced cells was 44.8% (CD176S-CAR-Ts) and 26.7% (CD176L-CAR-Ts), and these cells could be enriched to purities above 97% via EGFRt, which allowed direct comparison between CD176S- and CD176L-CAR-Ts (**Figure S3D**).

As expected, although the transduction and expansion protocol for the production of CD176-CAR-Ts involved cultivation in the presence of anti-CD3/CD28 beads for the first two days, all T-cell products remained CD176-negative during the whole manufacturing process (<0.6% CD176^+^ cells; **Figures S4A, B**). This suggests that activation and induction of cytokine release as part of CD176-CAR-T generation (**Figures S3C, E, F**) did not induce unmasking of CD176 on T cells, indicating their safety. Analogous to the assessment of CAR signaling in the reporter cell assay (**Figures 2B, C**), untransduced and CD176-CAR-transduced T cells were evaluated for activation after unmasking of CD176 following treatment with VCN. More than 96% of cells were CD176^+^ after two days of VCN treatment (**Figures S4C, D**). Evaluation of mean fluorescence intensity (MFI) of CD69 and CD137 by flow cytometry revealed equally low pre-activation of VCN-untreated CD176S- and CD176L-CAR-Ts when compared with untransduced T cells (**Figures S4E, F**). In contrast, unmasking of Sia-CD176 to CD176 by VCN resulted in increased expression of CD69 and CD137 on both CD176-CAR-Ts, which was significant for CD69 upregulation on CD176L-CAR-Ts. As additional control to decipher CD176 specificity, CD19-CAR-Ts were included in this experiment. Equally to untransduced T cells, the expression of CD69 and CD137 on these cells remained unchanged after VCN treatment (**Figures S4E, F**). However, VCN-induced activation of CD176-CAR-Ts did not result in fratricide as VCN treatment only slightly decreased the viability of CD176S- and CD176L-CAR-Ts in comparison to respective VCN-untreated CD176-CAR-Ts (data not shown). This was most likely due to critical impairment of formation of an immunological synapse, as VCN mediates the hydrolysis of all terminal sialic acids on the surface of treated cells and carbohydrates are known to be indispensable for key steps of anti-tumor T-cell functionality, including virtually all signaling cascades, T-cell activation, differentiation, proliferation, and interaction with tumor cells (27).

Thus, the ability of CD176-CAR-Ts to react towards CD176 was assessed by co-cultivation with identified CD176^-^ and CD176^+^ cell lines as models for various blood and solid cancer entities. Based on the results from the reporter assay data (**Figure 2D**), Loucy, A549, Panc-1 and NM-D4 were used as CD176^-^ and CD176^+^ target cells, respectively, for detailed analysis of CD176-CAR-T functionality.

**Figure 3:**
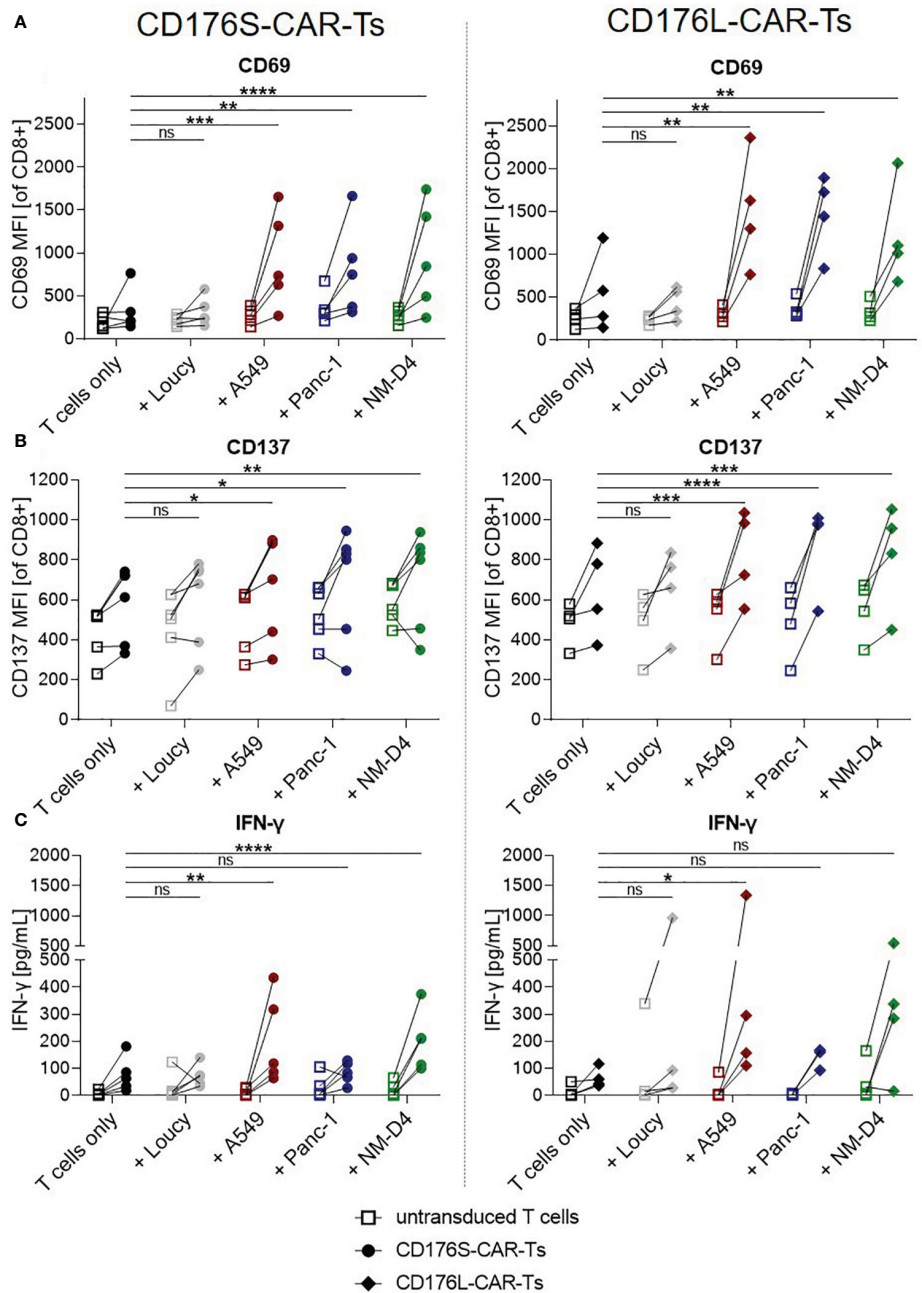
CD176-CAR-Ts are activated and release IFN-γ upon recognition of CD176^+^ cell lines from solid and blood cancer entities. CD176S- and CD176L-CAR constructs, respectively, were transduced into primary CD8^+^ T cells. After expansion and enrichment for CAR^+^ cells using EGFRt, T-cell products were either cultured alone (T cells only) or with the indicated target cells in an E:T ratio of 1:1. **(A, B)** After 24h, CD69 and CD137 mean fluorescence intensities (MFIs) on either CD176S- (left) or CD176L-CAR-Ts (right) were measured using flow cytometry. **(C)** Release of IFN-γ by either CD176S- (left) or CD176L-CAR-Ts (right) was measured after 48h by using LEGENDplex. **(A–C)** Symbols connected with one line represent data from one donor (n=3-6). Statistical analysis was performed using Two Way ANOVA and Tukey’s multiple comparisons tests, whereby only statistics to respective CD176-CAR-Ts only are shown. Statistical comparisons of co-cultures with untransduced T cells compared with untransduced T cells cultured alone were all not significant, except for the evaluation of CD137 upregulation on CD176S-CAR-Ts (**Figure 4B**, left), in which the control untransduced T cells co-cultured with NM-D4 cells expressed significantly more CD137 (**) than corresponding untransduced T cells cultured alone. ns, not significant, *p ≤ 0.05, **p ≤ 0.01, ***p ≤ 0.001, ****p ≤ 0.0001.

Significantly increased expression of CD69 and CD137 was determined on both, CD176S- and CD176L-CAR-Ts, after co-culturing for 24 hours with CD176^+^ target cell lines A549, Panc-1, and NM-D4 compared with the respective T cells cultured alone (**Figures 3A, B**). The activation was comparable for both constructs. Importantly, CD69 and CD137 MFIs of CD176S- and CD176L-CAR-Ts did not increase when co-cultured with CD176^-^ Loucy cells. Data from co-cultures for an extended period of time of 48 hours (**Figure S5**) confirmed the same pattern of target-specific T-cell activation.

To further characterize target-specific functionality of the CAR-Ts, co-culture supernatants were examined for secreted interferon-γ (IFN-γ) after 48h (**Figure 3C**). The release of IFN-γ was significantly increased by both CD176-CAR-Ts only after culture with A549 and, for the CD176S-CAR-Ts, also after recognition of NM-D4 cells when compared with the respective T cells only (mean release by CD176S-CAR-Ts: 205pg/mL (A549) and 202pg/mL (NM-D4) vs. 77pg/mL (T cells only), mean release by CD176L-CAR-Ts: 476pg/mL (A549) vs. 64pg/mL (T cells only)). As expected, co-culturing CD176-CAR-Ts with CD176^-^ Loucy cells did not trigger IFN-γ secretion.

Thus, both CD176-CARs were shown to mediate specific T-cell activation and IFN-γ release in a comparable extent upon recognition of CD176 on cancer cell lines as models for lung and pancreatic tumors and leukemia.

### 3.4 CD176-CAR-Ts mediate cytotoxicity and release effector molecules upon recognition of CD176^+^ cell lines from solid and blood cancer entities

**Figure 4:**
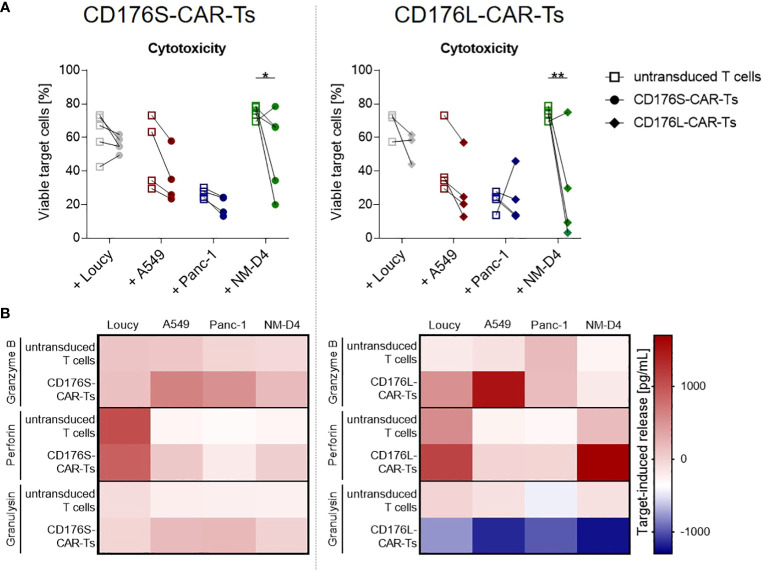
CD176-CAR-Ts mediate cytotoxicity and release effector molecules upon recognition of CD176^+^ cell lines from solid and blood cancer entities. CD176S- and CD176L-CAR constructs, respectively, were transduced into primary CD8^+^ T cells. After expansion and enrichment for CAR^+^ cells using EGFRt, T-cell products were co-cultured with the indicated target cells in an E:T ratio of 2:1 for 48h. **(A)** Reduction of previously CTV-labeled, viable target cells (7-AAD-negative cells) as indicator for cytotoxicity mediated by CD176S- (left) or CD176L-CAR-Ts (right) was measured by flow cytometry. Symbols connected with one line represent data from one donor (n=3-5). Statistical analysis was performed using Two Way ANOVA and Šídák’s multiple comparisons tests. *p ≤ 0.05, **p ≤ 0.01. **(B)** Release of granzyme B, perforin and granulysin by either CD176S- (left) or CD176L-CAR-Ts (right) was measured by using LEGENDplex. The concentrations measured in the corresponding T cells cultured in the absence of target cells were subtracted to obtain the target-induced release.

The cytotoxic capacity of CD176-CAR-Ts towards CD176^+^ cancer cells was further assessed by a flow cytometry-based cytotoxicity assay (**Figures 4A**; **S6**). Neither CD176-CAR-Ts mediated cytotoxicity against the CD176^-^ cell line Loucy after 48 hours of co-culture, but induced significantly reduced viability of CD176^+^ NM-D4 cells. In co-cultures of CD176-CAR-Ts with the CD176^+^ A549 cells, target cell viability was markedly but not significantly reduced in all individual donors compared with corresponding co-cultures with untransduced T cells. The same trend was observed when CD176S-CAR-Ts were co-cultured with CD176^+^ Panc-1 cells. Cytotoxicity measurements after an incubation period of 4 hours revealed the same pattern of CD176-CAR-mediated cell lysis of NM-D4 cells and even showed a significant reduction of viable Panc-1 cells by CD176L-CAR-Ts (**Figure S6**).

To confirm the specific cytotoxicity of CD176-CAR-Ts, the amount of released effector molecules granzyme B, perforin and granulysin was measured after 48 hours of co-cultivation with CD176^-^ and CD176^+^ cancer-cell lines (**Figure 4B**). After co-culture with CD176^-^ Loucy cells, CD176S-CAR-Ts did not release more cytotoxic effector molecules than untransduced T cells, but co-cultivation with CD176^+^ cell lines A549, Panc-1 and NM-D4 resulted in markedly higher concentrations of granzyme B, perforin and granulysin. CD176L-CAR-Ts co-cultured with CD176^-^ Loucy cells showed a slightly increased release of granzyme B and perforin compared with untransduced T cells, yet markedly increased release of granzyme B following co-cultivaton with the CD176^+^ cell line A549, as well as of perforin following co-culture with CD176^+^ NM-D4 cells.

### 3.5 CD176-CAR-Ts proliferate, release cytokines and mediate cytotoxicity in long-term co-cultures with CD176^+^ cells

**Figure 5:**
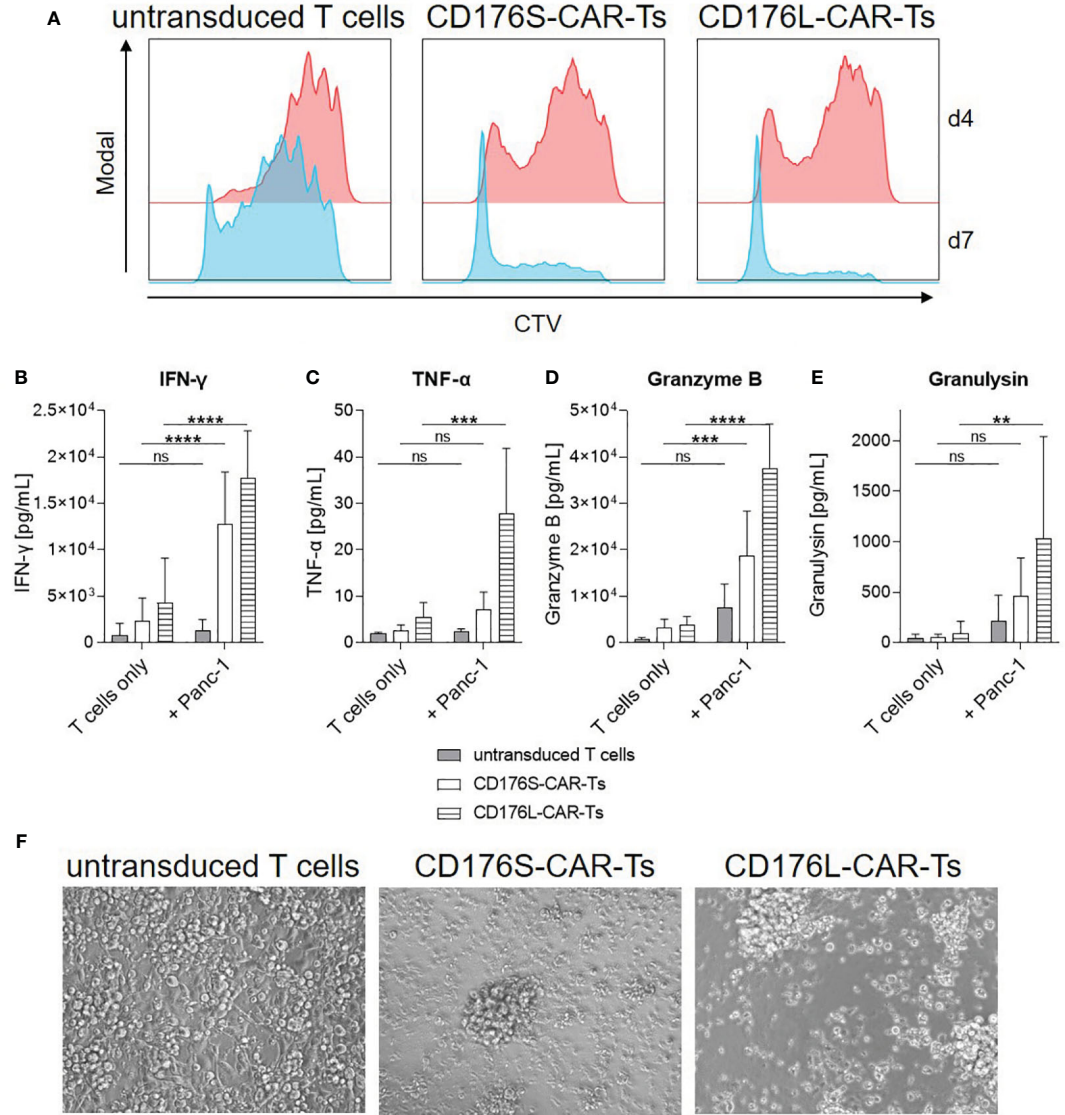
CD176-CAR-Ts proliferate, release cytokines and mediate cytotoxicity in long-term co-cultures with CD176^+^ cells. CD176S- and CD176L-CAR constructs, respectively, were transduced into primary CD8^+^ T cells. After expansion and enrichment for CAR^+^ cells using EGFRt, T-cell products were co-cultured with Panc-1 cells in an E:T ratio of 1:1 (n=5-6). **(A)** Proliferation of CTV-labeled T cells as assessed by flow cytometry after co-cultivation for four or seven days (d) is shown as representative dot plots. **(B–E)** Release of indicated soluble mediators was measured after co-cultivation for four days by using LEGENDplex and is shown as mean+SD. Statistical analysis was performed using Two Way ANOVA and Šídák’s multiple comparisons tests. ns: not significant. **p ≤ 0.01. ***p ≤ 0.001, ****p ≤ 0.0001. **(F)** After four days of co-cultivation, transmitted-light microscope images were taken with an Olympus IX81 microscope combined with a digital B/W camera using 10x objective lenses and analyzed with Xcellence Pro image software (all from Olympus). Representative pictures are shown.

To evaluate the proliferative capacity of CD176-CAR-Ts, they were co-cultured with CD176^+^ target cells for 4-7 days, whereby Panc-1 cells served as an exemplary solid tumor target cell line with intermediate CD176 expression. Already after 4 days, CD176S- and CD176L-CAR-Ts markedly proliferated in co-cultures with Panc-1 cells, whereby untransduced T cells only showed a low level of proliferation after an extended cultivation for 7 days (**Figure 5A**). Proliferation of CD176-CAR-Ts was accompanied by a target-specific and significantly increased release of different cytokines and effector molecules (IFN-γ, TNF-α, granzyme B and granulysin; **Figures 5B–E**). In that, CD176L-CAR-Ts released more cytokines when compared with CD176S-CAR-Ts. Moreover, in the long-term cultivation with Panc-1 for 4 days, distinct cytotoxicity by CD176S- and CD176L-CAR-Ts towards target cells was detectable via microscopical evaluation of adherent target cells (**Figure 5F**). In contrast, untransduced T cells did not lead to detachment of Panc-1 cells, nor the formation of cytotoxic clusters, indicating cytotoxicity observed for CD176S- and CD176L-CAR-Ts was CAR-mediated. Thus, exemplary shown for co-cultures with Panc-1 cells, CD176-CAR-Ts exhibited more pronounced cytotoxicity, cytokine and cytotoxic mediator release, and proliferation after an extended cultivation period of 4-7 days, whereby CD176L-CAR-Ts reacted slightly more than CD176S-CAR-Ts.

### 3.6 CD176-CAR-Ts proliferate and persist upon repetitive antigen stimulation

**Figure 6:**
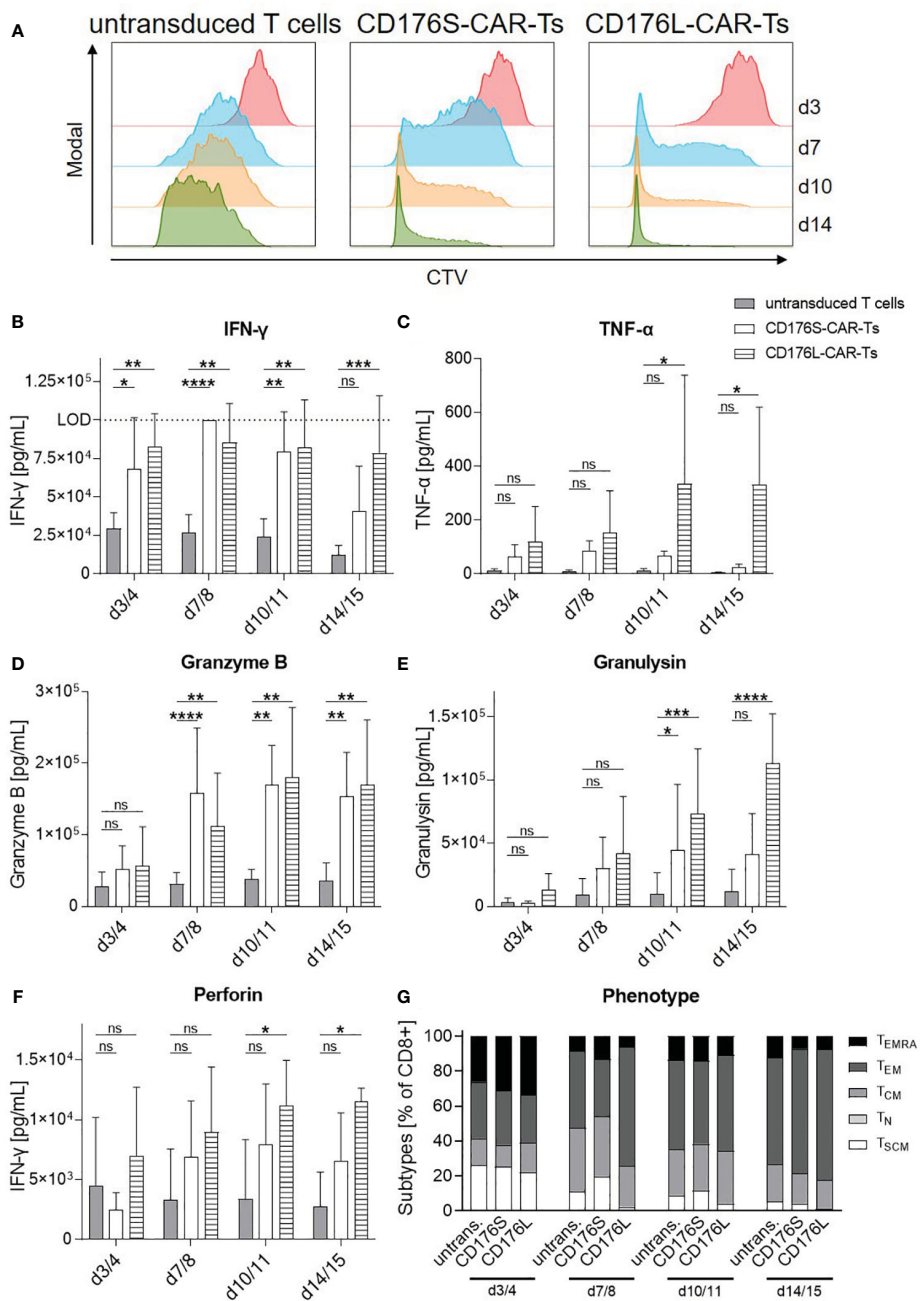
CD176-CAR-Ts proliferate and persist upon repetitive antigen stimulation. CD176S- and CD176L-CAR constructs, respectively, were transduced into primary CD8^+^ T cells. After expansion and enrichment for CAR^+^ cells using EGFRt, T-cell products were co-cultured with NM-D4 cells in an E:T ratio of 1:1. On day (d) 3/4, 7/8 and 10/11, all T cells were re-stimulated with the same number of target cells (n=3-4). **(A)** Proliferation of CTV-labeled T cells as assessed by flow cytometry is shown as representative dot plots. **(B–F)** Release of indicated soluble mediators as indicators for cytotoxicity towards NM-D4 cells was measured by using LEGENDplex and is shown as mean+SD. Statistical analysis was performed using Two Way ANOVA and Tukey’s multiple comparisons tests. ns: not significant. *p ≤ 0.05, **p ≤ 0.01. ***p ≤ 0.001, ****p ≤ 0.0001. **(G)** T-cell phenotypes during repetitive antigen stimulation were assessed by flow cytometry. Data is shown as mean. TEMRA: terminally differentiated effector memory T cells. TEM, effector memory T cells. TCM, central memory T cells. TN, naïve T cells. TSCM, stem-cell like memory T cells.

CD176-CAR-Ts were moreover evaluated for their proliferative capacity, persistence, and phenotype following repetitive antigen stimulation (**Figure 6**). For that, they were co-cultured with NM-D4 cells and re-stimulated for three times within two weeks, as NM-D4 cells mediated the most substantial stimulation of CD176-CAR-Ts in the evaluation of T-cell activation and cytotoxic capacity due to their high CD176 expression (**Figures 1C**, **3**, **4**). CD176S- and CD176L-CAR-Ts showed distinct and maintained proliferation during the whole experiment, whereas the proliferation of untransduced T cells was markedly slower (**Figure 6A**). In that, CD176L-CAR-Ts showed a slightly higher proliferative capacity compared with CD176S-CAR-Ts. Concomitantly, both CD176-CAR-Ts released significantly more cytokines and cytotoxic mediators than untransduced T cells in co-cultures with NM-D4 cells, indicating also target-specific and CAR-mediated cytotoxicity towards target cells (**Figures 6B–F**). For some of the soluble mediators, CD176L-CAR-Ts showed an increased secretion compared with CD176S-CAR-Ts. Importantly, although the cultivation medium was exchanged in every re-stimulation round, both CD176-CAR-Ts exhibited a stable or even increasing secretion over time, indicating persistence of highly-functional cells upon repetitive antigen stimulation. The phenotype of all T-cell products shifted towards an increased frequency of effector memory T (TEM) cells following repetitive stimulation, whereby phenotypes of CD176-CAR-Ts were not markedly different to respective phenotypes of untransduced T cells (**Figure 6G**). Thus, CD176-CAR-Ts are able to proliferate and persist upon repetitive antigen stimulation, whereby the functionality is maintained.

In conclusion, CD176 is a promising target for immunotherapy due to its broad occurrence on various blood and solid cancer types. CD176S- and CD176L-CAR-Ts efficiently recognize CD176 when exposed by VCN treatment or co-cultured with CD176^+^ target cells. Recognition of the target leads to specific activation, target cell lysis, release of cytokines and cytotoxic mediators, and proliferation, particularly when co-cultured with CD176^+^ A549, Panc-1 and NM-D4 cells. In that, their functionality was maintained upon repetitive stimulation with target cells for two weeks. Importantly, CD176S- and CD176L-CAR-Ts are neither activated nor mediate effector functions towards CD176^-^ control cells, demonstrating their CD176-specific functionality. Our data indicate that the short spacer construct in CD176S-CAR-Ts, compared with the CD176L-CAR-Ts, allows for increased capacity of CAR signaling, including response towards two cell lines that were not recognized by CD176L-CAR-Ts. In line, CD176S-CAR-Ts tended to be less preactivated and exhibited a more CD176-specific cytokine release. Nevertheless, both generated CD176-CAR-T products were activated and mediated cytotoxicity towards CD176^+^ cancer cells in an equivalent and target-specific manner, and CD176L-CAR-Ts showed an even slightly increased functionality in long-term and repetitive co-cultures with target cells.

## 4 Discussion

We investigated the specificity and functionality of CAR-Ts directed against the TF antigen CD176. CD176 was confirmed to be detectable on tumor sections of lung adenocarcinoma, breast, pancreatic, head and neck, esophagus and ovarian cancer patients, as well as on leukemic cells of T- and B-ALL patients by using Nemod-TF2. Among leukemia patients, the frequency of CD176^+^ malignant cells was higher in patients with a relapse, which is in line with the study of Eldesouki et al. (15) which showed that quiescent leukemia stem cells associated with disease progression and therapy resistance express CD176 in CML patients. As models for a variety of the identified CD176^+^ blood and solid cancer entities, the cell lines A549 (lung cancer), Panc-1 (pancreatic cancer), HL-60 and NM-D4 (leukemia) as well as MCF-7 (breast cancer) were found to be CD176-positive. Importantly, on healthy cells, CD176 is extended by carbohydrate chains or terminal sialylation and thus virtually unavailable for antibody binding. In our study, CD176 was not detected on most healthy tissue sections or on PBMCs from healthy donors. In two out of seven normal lung tissue samples from different donors, a positive CD176 staining was detected in the alveolar epithelium. As this is opposed to findings by others that proved absence of CD176 on various glandular and epithelial tissues from trachea and lung (12), presence of CD176 on healthy cells and their potential recognition by CAR-Ts should be carefully examined in future safety studies. Nemod-TF2 is described to have similar binding properties to the established anti-CD176 antibody A78-G/A7 with particularly high affinity and specificity, but equal small cross-reactivity to core-2 and, to some extent, to the β-anomeric configuration of Galβ1-3GalNAc (9). The majority of CD176-specific antibodies share these cross-reactivities, and neither structure was found to be detectable in an unmasked version in healthy or malignant tissues. Consistent with this, Cao et al. proved cells from a wide range of healthy tissues to lack detectable CD176 by staining with A78-G/A7 (12). As the only healthy tissues, Cao et al. found A78-G/A7 binds to few luminal membranes of pancreatic ducts, distal renal tubules and collecting ducts, the cytoplasm of some macrophages, spermatids and the placenta (12). These structures should be included in safety studies, although most of them are immunopriviledged sites or unlikely to be reached by CAR-Ts. Moreover, CD176 expression in non-tumoral tissues, e.g. during inflammation should be further investigated, as a few studies showed increased expression of CD176 in human mucosal samples from chronic inflammatory bowel disease (reviewed in 28, 29). However, in a cotton-top tamarin model, these higher levels of CD176 expression were significantly associated with the development of colorectal carcinoma, suggesting a possible role in cancer development rather than a ubiquitous mechanism during inflammation. In our study, despite considerable activation and cytokine release induced in untransduced and CD176-CAR-transduced T cells by anti-CD3/CD28 beads as part of their manufacturing process, CD176 was not unmasked, which supports absence of CD176 in this immune process and indicates safety.

In most healthy individuals, low levels of natural IgM antibodies against CD176 can be detected, which are hypothesized to be induced by gastrointestinal bacteria carrying cross-reacting carbohydrate structures (30). In gastric cancer patients, high levels of natural anti-CD176 antibodies were moreover shown to be associated with a benefit in survival (31). Different vaccination studies were conducted to boost levels of CD176-specific antibodies in the blood of carcinoma patients. For instance, MacLean et al. demonstrated in a clinical study that ovarian cancer patients produced IgM antibodies, followed by IgG and IgA after immunization with a synthetic CD176 antigen (32). However, the majority of these antibodies were only specific against synthetic CD176 antigen and failed to recognize CD176 on natural ligands.

Thus and based on their multivalent nature and potentially decade-long persistence in patients (3), in the present study, we generated CAR constructs and the corresponding T cells targeting CD176 based on the scFv of Nemod-TF2 as novel immunotherapy approach. CD176-CAR-Ts specifically recognized CD176 present on tumor cell lines or unmasked by VCN treatment as indicated by T-cell signaling and activation. For VCN-treated CD176-CAR-Ts, this activation was not translated to significant fratricide, most likely due to critical impairment in the formation of an immunological synapse by hydrolysis of all terminal sialic acids on the surface of CD176-CAR-Ts, as glycans are described to be essential for various key steps of anti-tumor T-cell functionality (33). However, specific recognition of CD176 on the surface of tumor cell lines resulted in proliferation, target cell lysis, and release of cytokines and cytotoxic mediators particularly in response to recognition of CD176^+^ A549 (lung adenocarcinoma), Panc-1 cells (pancreatic epithelioid carcinoma), and NM-D4 (CML) cells. As mentioned above, one of the biggest challenges in the choice of target is to avoid “on-target/off-tumor toxicities”, i.e. to avoid elimination of healthy cells. For example, in a study investigating CAR-Ts against carboxy-anhydrase-IX for the treatment of metastatic renal cell carcinoma, high-grade liver toxicities were observed, which were due to low expression of the CAR target on healthy liver cells (7). In addition, a dramatic case was reported, in which a patient with metastatic colon carcinoma was treated with anti-ERBB2-CAR-Ts and subsequently died of respiratory failure, which was attributed to “on-target/off-tumor” toxicities in the lung (6). In the present study, the specificity of CD176 detection is particularly demonstrated by the fact that there was no T-cell response to the CD176^-^ target cell line Loucy and PBMCs, so that the likelihood of “on-target/off-tumor” cytotoxicity for CD176 as a target can be considered very low. Solely, evaluation of a potential background reactivity against normal lung tissue with low detectable CD176 levels should, as mentioned above, included in future safety studies.

Since the distance between CAR and target epitope is crucial for recognition and thus CAR functionality (23) and the membrane proximity of CD176 is dependent on its carrier molecules being different on each individual cell type (11, 14, 34), two CD176-CAR constructs differing in the length of their spacer domain were studied. CD176 is always expressed in context of carrier proteins and most frequently on mucins of epithelial cells. Multiple molecules carrying CD176 are described for different types of malignancies. These include MUC1 and CD44 for breast cancer (14), CD44 for colon cancer (34), or CD34 for leukemia (11), although one cell is not restricted to only one carrier. For example, Lin et al. proved the co-expression of cancer initiation cell (CIC) markers CD44 and CD133 with CD176 on lung, breast, and liver cancer (14).

For most cancer entities tested in the present study, the CD176S-CAR mediated equal or improved target-cell recognition compared with the CD176L-CAR. These findings suggest that CD176 is present on these cells in a sterically similar configuration, and this can be optimally bound by a short-spacer CAR construct. CD176S-CARs weakly recognized the CD176^+^ APL cell line HL-60 and the CD176^+^ breast adenocarcinoma cell line MCF-7, while CD176L-CARs did not respond to both cell lines. Thus, the question arises whether A549, NM-D4 and Panc-1 have common carrier molecules for CD176 with optimal binding properties for CD176-CARs that are absent on MCF-7 and HL-60. Another explanation for the differential CD176-CAR activation could be that some cell lines with MUC1 as CD176 carrier are known to exhibit immunoinhibitory features, e.g. by binding of ICAM-1 on T cells, which can induce T-cell anergy (35). Whether this mechanism also applies to the cell lines MCF-7 and HL-60 has not been investigated. On the other hand, however, MUC1 expressed on NM-D4 cells was not found to be immunoinhibitory (36), possibly explaining the strong induction of CAR-mediated effector functions by these cells. Agrawal et al. found that addition of IL-2 or CD28-targeting monoclonal antibodies could reverse the MUC1-induced T-cell suppression (35), which would be interesting to investigate in future studies. Whereas the variety of recognized tumor cell lines was broader for CD176S-CAR-Ts, CD176L-CAR-Ts showed a slightly higher proliferative capacity and cytokine response towards recognized target cells following long-term or repetitive stimulation with target cells indicating that the receptor design is more favorable for maintaining T-cell function over time.

Within an individual tumor tissue or cell line, CD176 was expressed in a varying proportion of malignant cells, which has been attributed to a fluctuating CD176 expression over time not uncommon for carbohydrate antigens (9). In line, CD176^high^ and CD176^-/low^ cell fractions of CD176^+^ cell lines returned to similar CD176 levels after a short time of cultivation, suggesting that individual cells within each cell line express CD176 to varying and fluctuating levels, rather than that some tumor cells do not unmask CD176 at all. Since 4-1BB was used as costimulation domain of CD176-CAR constructs, and the majority of generated CD176-CAR-Ts showed a CD8^+^ TCM phenotype, both of which are known to mediate long-term persistence *in vivo* (37, 38), we expect a CD176-targeting immunotherapy to reach a majority of cancer cells of an individual cancer over time. In line, CD176-CAR-Ts markedly proliferated upon repetitive antigen stimulation with CD176^+^ target cells and maintained their functionality over a time period of two weeks. In a future study, usage of CD176-CAR-transduced CD4^+^ and CD8^+^ in a ratio of 1:1 will potentially even increase persistence, as CAR-transduced CD4^+^ T cells were shown to produce more type 1 cytokines and exhibit higher proliferative potential compared to CAR-transduced CD8^+^ T cells and mediate synergistic antitumor activity with CAR-transduced CD8^+^ T cells (39). The mechanism by which CD176 is exposed on malignant cells has not yet been fully understood. Aberrant glycosylation is a key feature of cancers, and several alterations of the enzymatic activities of the monosaccharide transferases system, nucleotide transporters and epimerases, as well as an increased transit rate of CD176-carrying proteins to the surface are thought to be involved (9). Detection of CD176 on the surface of malignant cells has been shown to correlate with disease progression, invasion and metastasis (9, 13–15, 40). For example, a study examining colorectal carcinoma sections revealed that CD176 was significantly more abundant on liver metastases from colorectal cancer compared to the primary tumor and that the risk of metastasis of CD176^+^ primary tumors was significantly increased compared with CD176^-^ tumors (57% vs. 14%) (40). The interaction of CD176 on circulating cancer cells and Galectin-3 expressed on the endothelium of distant organ vessels were found to mediate the initial, rate-limiting stages of hematogenous metastasis in several cancer entities, including breast, prostate, colon and pancreatic carcinomas (41). Therefore, we hypothesize that CD176-CAR-Ts eliminate the potentially most harmful cancer cells with high metastatic potential by targeting CD176. Moreover, CAR targets with an important role in growth and survival of cancer cells were shown to be less prone for downregulation and loss-of-function (42), suggesting that the probability of antigen escape should be low for CD176-CAR-Ts.

While successful in the treatment of hematological malignancies (3), the development of CAR-Ts for the treatment of solid tumors is challenged by multiple factors including their immunosuppressive microenvironment limiting T-cell function (8). To improve cytotoxicity of CD176-CAR-Ts, development of 4th generation CD176-CAR-Ts, also known as TRUCKs (T cells redirected for universal cytokine-mediated killing), which enable localized secretion of a pro-inflammatory cytokine such as IL-12 or IL-18 upon target recognition (43), might be an attractive strategy. Depending on the choice of cytokine, this strategy is expected to improve T-cell functionality and, most importantly, attract additional kinds of immune cells and remodel the tumor microenvironment of solid tumors (22). By that, also target-negative tumor cells can be approached by tumor-specific innate immune cells, which would otherwise be inaccessible by conventional CAR-T therapy (44). Especially with regard to heterogeneity and fluctuation of CD176 expression on cancer cells, the TRUCK approach is interesting but particularly needs to be investigated for potential toxicities. As a further step, it is important to test whether CD176-specific CAR-Ts are able to effectively control cancer growth and metastasis *in vivo*, whereby combination with other CARs or bispecific T-cell engagers, as tested for other targets (reviewed in 45), would be a potential strategy to further increase efficacy.

The results of our study demonstrate for the first time that the prominently expressed carbohydrate cancer antigen CD176 can be targeted with CAR-T technology. We could show that CD176-CAR-Ts are promising candidates in that they may indeed be suitable for eradication of malignant cells from multiple blood or solid cancer entities while reducing common CAR-associated side effects. Furthermore, we showed that the choice of the correct spacer length has a crucial impact on the recognition and persistence of genetically-modified effector cells and speculated that a broad endowment of inducible cytokines may improve for effective therapy.

## Data availability statement

The original contributions presented in the study are included in the article/**Supplementary Material**. Further inquiries can be directed to the corresponding author.

## Ethics statement

The human samples used in this study were acquired from residual blood samples from routine platelet collection at the Institute of Transfusion Medicine and Transplant Engineering, Hannover Medical School. Written informed consent was obtained from all donors as approved by the Ethics Committee of Hannover Medical School (2519–2014, 3639–2017).

## Author contributions

Conceptualization, AD, LB, AB, SR, FY, RB, MHus, MHud, BE-V; methodology, LB, AD, MU, CM, LR, PK, JG, KZ; software, LB, AD, MU, AB, LR, BE-V; validation, AD, LB, AB, BE-V; formal analysis, LB, AD, AB; investigation, AD, LB, MU, AB, CM, PK, JG, TL, LW, SM-H, BE-V; resources, RB, PK, JG, LW, SM-H, FT, GB, BM-K, AS, BE-V; data curation, LB, AD, AB, BE-V; writing—original draft preparation, AD, LB, AB, BE-V; writing—review and editing, all authors; visualization, AD, LB; supervision, AD, AB, BM-K, MHud, BE-V; project administration, BE-V; funding acquisition, AD, SR, RB, MHus, BE-V. All authors contributed to the article and approved the submitted version.
