# Supplementary material for: CAR-Ts redirected against the Thomsen-Friedenreich antigen CD176 mediate specific elimination of malignant cells from leukemia and solid tumors
Source: Front Immunol. 2023 Oct 17;14:1219165. doi: 10.3389/fimmu.2023.1219165 (PMC10616308; doi:10.3389/fimmu.2023.1219165)
Supplement: Supplementary file 1 [file DataSheet_1.docx]

Supplementary Material

CAR-Ts redirected against the Thomsen-Friedenreich antigen CD176 mediate specific elimination of malignant cells from leukemia and solid tumors

Anna Christina Dragon^†^, Luca Marie Beermann^†^, Melina Umland, Agnes Bonifacius, Chiara Malinconico, Louisa Ruhl, Patrik Kehler, Johanna Gellert, Lisa Weiß, Sarah Mayer-Hain, Katharina Zimmermann, Sebastian Riese, Felicitas Thol, Gernot Beutel, Britta Maecker-Kolhoff, Fumiichiro Yamamoto, Rainer Blasczyk, Axel Schambach, Michael Hust, Michael Hudecek and Britta Eiz-Vesper*

^†^These authors have contributed equally to this work and share first authorship

***Correspondence:** Prof. Dr. Britta Eiz-Vesper: eiz-vesper.britta@mh-hannover.de

# Supplementary Figures and Table


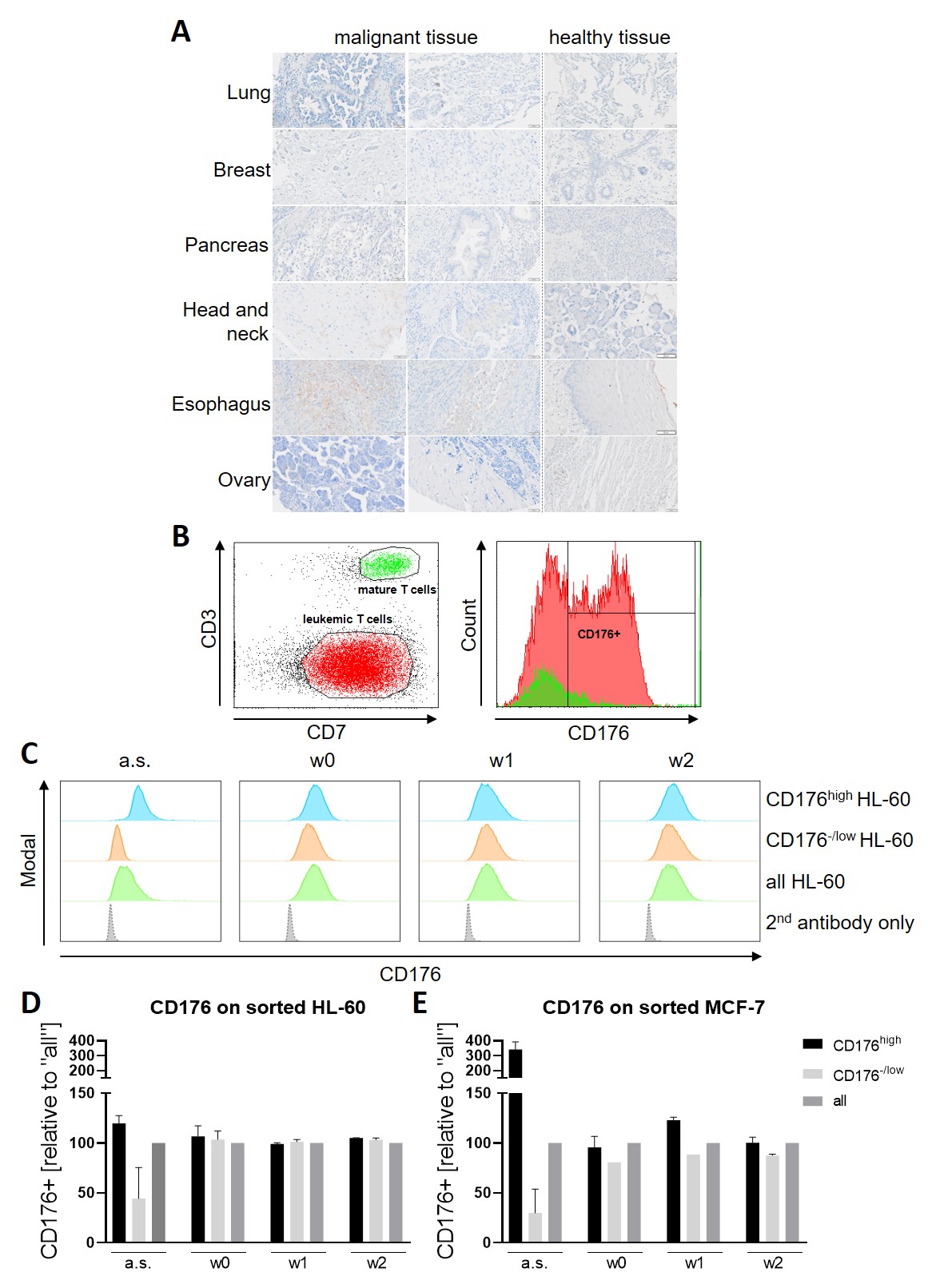


**Figure S1: Detection of CD176 in solid tumor and T-ALL patients, and on cell lines as surrogates. (A)** Tissue samples of healthy donors or patients with different kinds of cancer (healthy tissue array and tumor tissue array) were stained by immunohistochemistry using a murine IgM isotype control to confirm CD176 specificity. All slides were counterstained using Mayer’s Haematoxylin. **(B)** Exemplary, CD176 expression on PBMCs isolated from a T-ALL patient was evaluated on leukemic cells gated as CD3^-^CD7^+^ and mature T cells as CD3^+^CD7^+^ by using murine Nemod-TF2 and flow cytometry. **(C-D)** HL-60 and **(E)** MCF-7 cells were sorted into a cell fraction with high CD176 expression (CD176^high^) and a fraction with low or absent CD176 expression (CD176^-/low^). Corresponding cells sorted for all viable cells (all) served as controls. Presence of CD176 was detected immediately after sorting (a.s.) and in the following weeks (w0 = until 7 days a.s., w1: 8-14 days a.s., w2: 15-21 days a.s.) by flow cytometry, and is shown as **(C)** representative histograms or **(D)** mean+SD relative to “all” (n=2-3).


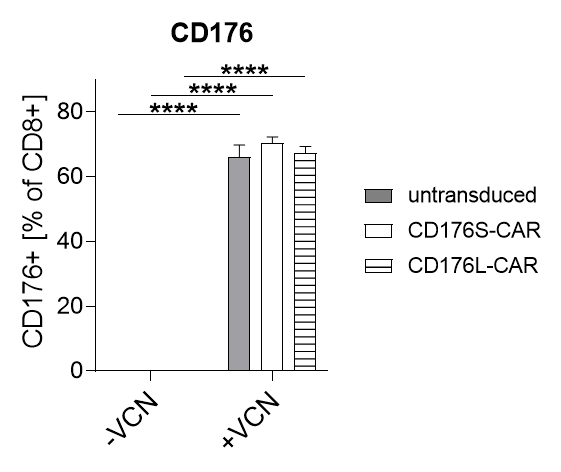


**Figure S2: Unmasking of terminal CD176 on CD176-CAR-transduced reporter cells.** CD176-CAR-transduced reporter cells were treated with VCN (+VCN) or left untreated (-VCN). After two days, expression of CD176 was measured by staining with Nemod-TF2 and flow cytometry (n=5). Data are shown as mean+SD. Statistical analysis was performed using two-way ANOVA and Šídák's multiple comparisons test. ****p≤0.0001.


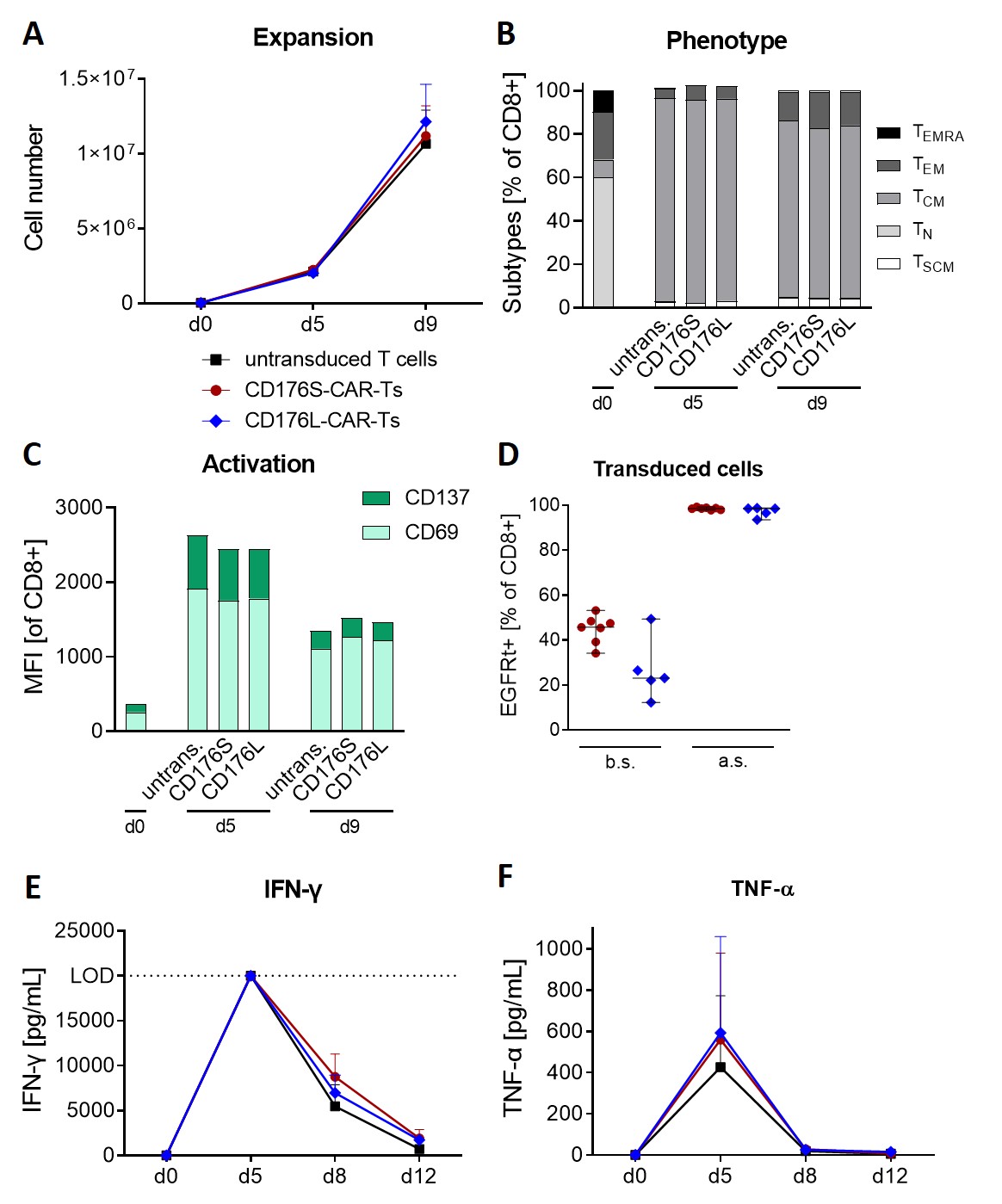


**Figure S3: Generation of CD176-CAR-Ts.** CD176S- and CD176L-CAR constructs, respectively, were transduced into primary CD8^+^ T cells. **(A)** Cell numbers (n=6-8), as well as **(B)** phenotype and **(C)** expression of CD69 and CD137 as assessed by flow cytometry during the initial expansion (n=6). **(D)** On day 8-9, transduced T cells were enriched by using EGFRt as selection marker. Frequency of transduced cells was assessed before sorting (b.s.) and after sorting (a.s.) by staining for EGFRt and flow cytometry (n=5-7). **(E, F)** Release of cytokines during CAR-T manufacturing was assessed by LEGENDplex (n=4). Data are shown as **(A, D-F)** mean±SD or **(B, C)** mean. TEMRA: terminally differentiated effector memory T cells. TEM: effector memory T cells. TCM: central memory T cells. TN: naïve T cells. TSCM: stem-cell like memory T cells. LOD: limit of detection.


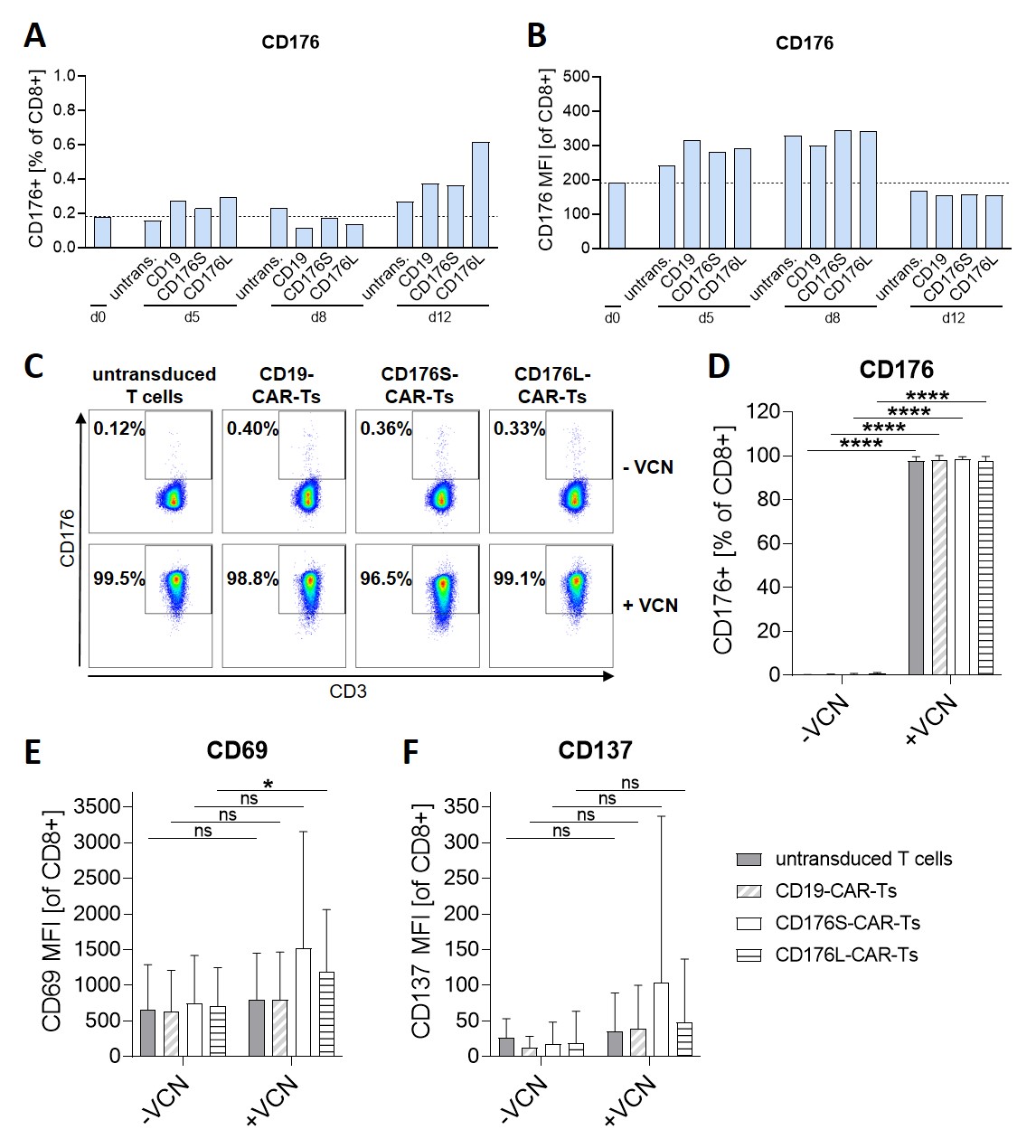


**Figure S4: Expression and umasking of terminal CD176 on CD176-CAR-Ts.** CD176S- and CD176L-CAR constructs, respectively, were transduced into primary CD8^+^ T cells, expanded and CAR^+^ cells enriched on day 8-9 using EGFRt. Untransduced T cells and CD19-CAR-Ts generated analogously served as controls. **(A, B)** CD176 expression was assessed during manufacturing of CD176-CAR-Ts and is shown as **(A)** mean frequency and **(B)** mean MFI (n=4). **(C-F)** After expansion, T-cell products were either treated with VCN (+VCN) for 48h or left untreated
(-VCN). Expression of **(C, D)** CD176 as **(C)** representative dot plots and **(D)** mean frequencies, as well as mean fluorescence intensities (MFIs) of **(E)** CD69 and **(F)** CD137 were analyzed by flow cytometry. **(D-F)** Data are shown as mean+SD (n=5-6). Statistical analysis was performed using two-way ANOVA and Šídák's multiple comparisons test. ns: not significant, *p≤0.05, ****p≤0.0001.


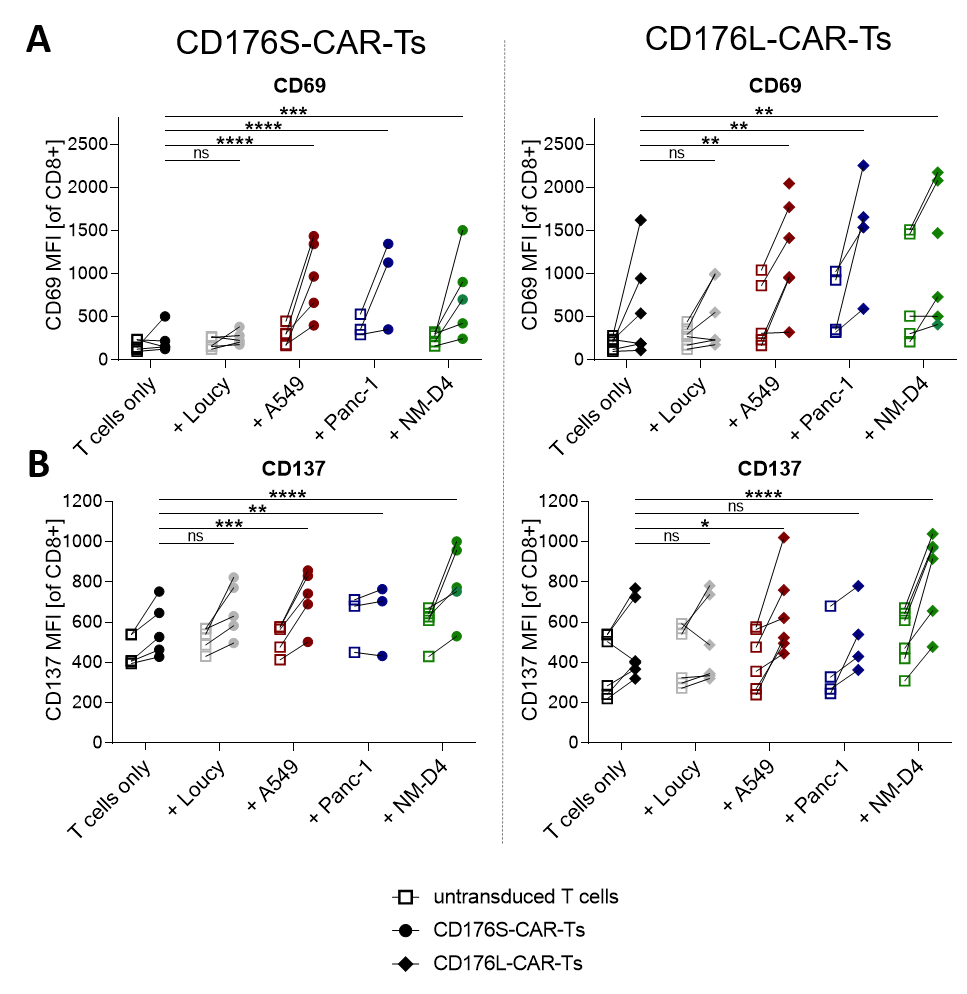


**Figure S5: CD176-CAR-Ts are activated upon recognition of CD176^+^ cell lines from solid and blood cancer entities.** CD176S- and CD176L-CAR constructs, respectively, were transduced into primary CD8^+^ T cells. After expansion and enrichment for CAR^+^ cells using EGFRt, T-cell products were either cultured alone (T cells only) or with the indicated target cells in an E:T ratio of 1:1 for 48h. **(A)** CD69 and **(B)** CD137 mean fluorescence intensities (MFIs) on either CD176S- (left) or CD176L-CAR-Ts (right) were measured using flow cytometry (n=4-6). Statistical analysis was performed using Two Way ANOVA and Tukey’s multiple comparisons tests, whereby only statistics to respective CD176-CAR-Ts only are shown. Symbols connected with one line represent data from one donor. ns: not significant, *p≤0.05, **p≤0.01, ***p≤0.001, ***p≤0.0001.


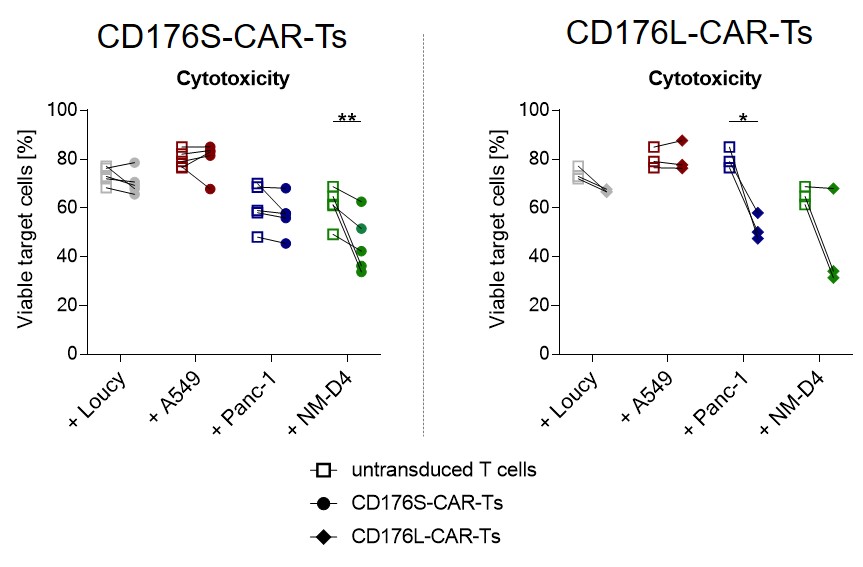


**Figure S6: CD176-CAR-Ts are mediate cytotoxicity upon recognition of CD176^+^ cell lines from solid and blood cancer entities.** CD176S- and CD176L-CAR constructs, respectively, were transduced into primary CD8^+^ T cells. After expansion and enrichment for CAR^+^ cells using EGFRt, T-cell products were cultured with the indicated, previously CTV-labeled target cells in an E:T ratio of 2:1 for 4h. Reduction of viable target cells (7-AAD-negative cells) as indicator for cytotoxicity mediated by CD176S- (left) or CD176L-CAR-Ts (right) was measured by flow cytometry (n=3-5). Statistical analysis was performed using Two Way ANOVA and Šídák's multiple comparisons tests. Symbols connected with one line represent data from one donor. ns: not significant, *p≤0.05, **p≤0.01.

**Table S1. Presence of CD176 on tumor samples from head and neck cancer and esophagus cancer patients.** Tissue samples of patients with head and neck cancer and esophagus cancer (tumor tissue array) were stained using Nemod-TF2. All slides were counterstained using Mayer’s Haematoxylin and evaluated by two independent analysts to determine the immunoreactive score (IRS) for presence of membranous CD176 on tumors.

|  | **Head and neck cancer** | **Esophagus cancer** |
| --- | --- | --- |
| **IRS = 0** | 2 | 6 |
| **IRS = 1-3** | 5 | 3 |
| **IRS = 4-8** | 3 | 8 |
| **IRS = 9-12** | 0 | 1 |
| **% positive** | 80% | 67% |

# Supplementary Methods

# Cell lines

Cell lines used and their respective cultivation medium are listed in table S1. Cell lines used in target cell assays were stained for CD176 using a humanized anti-CD176 IgM antibody derived from Nemod-TF2 [9] (Glycotope) as a primary antibody. Staining was carried out before each functional assays to maintain reliable results. The CD176^-^ cell line Loucy served as a negative control line. The cell line NM-D4 is a selection of CD176-expressing K562 cells co-expressing MUC1 as well Glycophorin [36].

**Table S2.** DMEM (Lonza), Fetal bovine serum (FBS; Sigma-Aldrich), L-glutamine (c.c.pro), RPMI 1640 medium (Lonza).

| **Cell line** | **Cultivation medium** | **Order Number** | **Supplier** |
| --- | --- | --- | --- |
| 293T | DMEM, 10% FBS, 2 mM L-glutamine | ACC-635 | DSMZ |
| A549 | DMEM, 10% FBS, 2 mM L-glutamine | ACC-107 | DSMZ |
| HL-60 | RPMI 1640, 10% FBS, 2 mM L-glutamine | ACC-3 | DSMZ |
| JE6-1 reporter | RPMI 1640, 10% FBS, 2 mM L-glutamine |  | Kindly provided by Prof. Steinberger [24] |
| Jurkat | RPMI 1640, 10% FBS, 2 mM L-glutamine | ACC-282 | DSMZ |
| Loucy | RPMI 1640, 10% FBS, 2 mM L-glutamine | ACC-394 | DSMZ |
| MCF-7 | DMEM, 10% FBS, 2 mM L-glutamine | ACC-115 | DSMZ |
| NM-D4 | RPMI 1640, 10% FBS, 2 mM L-glutamine | ACC-2605 | Glycotope |
| Panc-1 | DMEM, 10% FBS, 2 mM L-glutamine | CRL-1469 | ATCC |

# Generation and titration of lentiviral vectors

CD176S and CD176L CAR lentiviral particles were produced in 293T (DSMZ) cells using the calcium phosphate method as described before [22]. Briefly, 293T cells were transfected with respective CD176-CAR-epHIV7 vectors and psPAX2 and pMD2.G second-generation packaging vectors in presence of 25 µM chloroquine. Supernatants were harvested after 32h and 48h and concentrated via centrifugation at 10000xg for 16h at 4 °C. In order to determine the titers of viral supernatants, Jurkat cells (DSMZ) were transduced in the presence of 5 µg/ml Polybrene Infection/Transfection Reagent. The transduction efficiency was assessed after 48h by staining the transduced cells with biotin-anti-EGFRt (biotin-labeled Erbitux; ImClone Systems) and PE streptavidin (Thermo Fisher Scientific), and using flow cytometry.

# Flow cytometry

Antibodies used for flow cytometry are listed in table S2. CD176 was detected by using the humanized version of mAb Nemod-TF2 (Glycotope) and anti-human IgG+IgM secondary antibody (Jackson ImmunoResearch), except for the staining of PBMCs, which was carried out by using the murine mAb Nemod-TF2 and anti-mouse Ig (BD Biosciences) to avoid background staining on human cells. The anti-EGFRt antibody was obtained (Erbitux; ImClone Systems) and coupled to biotin (Thermo Fisher Scientific) for further use.

**Table S3.** Antibodies used for flow cytometry. Peridinin-chlorophyll-protein (PerCP), Alexa Fluor® (AF), allophycocyanin (APC), Brilliant Violet^TM^ (BV), phycoerythrin (PE), fluorescein isothiocyanate (FITC).

| **Specificity** | **Antibody Clone** | **Fluorophore** | **Supplier** |
| --- | --- | --- | --- |
| CD3 | SK7 | PerCP or AF700 | BioLegend |
| CD3 | SK7 | PerCP-Cy5.5 | eBioscience |
| CD7 | M-T701 | APC | BD Pharmingen |
| CD8 | SK1 | AF700 or BV510 | BioLegend |
| CD10 | HI10a | PerCP-Cy5.5 | BioLegend |
| CD19 | HIB19 | APC | BD Biosciences |
| CD34 | 581 | PE-Cy7 | BD Biosciences; BioLegend |
| CD45 | 2D1 | APC-H7 | BD Biosciences |
| CD45RO | UCHL1 | APC-Cy7 | BioLegend |
| CCR7 | G043H7 | AF700 | BioLegend |
| CD95 | DX2 | BV421 | BioLegend |
| CD69 | FN50 | BV605 | BioLegend |
| CD137 | 4B4-1 | APC | BioLegend |
| EGFRt | - | (biotin) | ImClone Systems |
| Streptavidin | - | PE | Thermo Fisher Scientific |
| CD176 | Nemod-TF2-derived hIgM | - | Glycotope |
| Human IgG+IgM | - | PE or FITC | Jackson ImmunoReserach |
| human IgM | MHM-88 | FITC | BioLegend |
| CD176 | Nemod-TF2-mIgM | - | Glycotope |
| Mouse Ig | gIg | PE | BD Biosciences |
